# Member selection in traditional Chinese medicine innovation consortia: an evolutionary game theory approach

**DOI:** 10.3389/fmedt.2026.1786671

**Published:** 2026-07-02

**Authors:** Dong Hua, Yanhui Wang

**Affiliations:** 1 School of Health Economics and Management, Nanjing University of Chinese Medicine, Nanjing, China; 2 School of Elderly Care Services and Management, Nanjing University of Chinese Medicine, Nanjing, China

**Keywords:** collaborative innovation, evolutionary game, innovation consortia, member selection, third-party involvement, traditional Chinese medicine (TCM)

## Abstract

**Introduction:**

In recent years, long and uncertain research and development (R&D) cycles, difficulties in patent protection, and fragmented innovation activities have jointly constrained the traditional Chinese medicine (TCM) industry, making many enterprises hesitant to pursue independent innovation. Despite a relatively complete industrial chain, traditional Chinese medicine (TCM) industry faces persistent challenges in technological innovation and commercialization. Collaborative innovation through TCM innovation consortia has emerged as a strategic mechanism to integrate heterogeneous resources and enhance innovation capacity. Understanding the determinants of member selection and consortium formation dynamics is essential for promoting high-quality development.

**Methods:**

This study develops an evolutionary game model under bounded rationality to simulate potential consortium members’ decision-making. Key factors—resource complementarity, transaction costs, participant strengths, and third-party involvement—are incorporated to evaluate their impact on stable consortium formation.

**Results and discussion:**

Analysis indicates that higher resource complementarity, balanced participant strengths, greater expected returns, and third-party support significantly increase the likelihood of stable consortium formation. Similar mechanisms are observed in international innovation ecosystems, where intermediaries, policy guidance, and cross-boundary partnerships reduce information asymmetries and transaction costs. These findings offer theoretical insights into dynamic member selection and practical guidance for enhancing governance, operational stability, and coordination efficiency in TCM innovation consortia. By learning from global collaboration practices, more effective resource integration and cooperation can strengthen innovation capacity and support the high-quality development of China's TCM industry.

## 1 Introduction

The challenges facing the traditional Chinese medicine (TCM) innovation system extend beyond isolated technical or institutional constraints and reflect deeper behavioral dynamics. Long and uncertain research and development (R&D) cycles, persistent difficulties in protecting knowledge assets—particularly classical formulas and accumulated clinical experience—and fragmented innovation activities across organizations jointly shape an environment characterized by high uncertainty and weak coordination.

These challenges are not unique to the TCM sector, but are widely observed in innovation systems based on traditional and indigenous knowledge. Such knowledge systems are often characterized by strong tacitness, collective ownership attributes, and difficulties in formal intellectual property protection, which complicate both knowledge governance and innovation incentives (1). As a result, designing effective collaborative mechanisms becomes particularly critical in these contexts.

More importantly, these constraints are not independent. They interact to reinforce coordination failures and undermine incentives for sustained collaboration among firms, research institutes, and hospitals. As a result, improving innovation performance in the TCM sector requires not only addressing individual barriers, but also urgently understanding how collaborative arrangements can be structured to mitigate uncertainty and align incentives across heterogeneous actors.

As one of the few sectors in China with an indigenous technological foundation and long-standing innovative potential, the TCM industry has developed a relatively complete industrial chain, encompassing upstream cultivation and breeding of medicinal materials, midstream processing of TCM decoction pieces and manufacturing of proprietary Chinese medicines, and downstream wholesale and retail distribution. Despite this structural completeness, the overall low level of industrial concentration in China's TCM sector (with most firms operating on a small scale) means that the industry suffers from insufficient technological innovation, extensive and inefficient production methods, and a low rate of commercialization of research outputs.

Under such conditions, collaborative innovation has emerged as a necessary pathway for enhancing technological capabilities. In innovation research, multi-actor alliances such as R&D consortia have long been identified as important mechanisms through which heterogeneous organizations share risks, integrate resources, and generate innovation outputs beyond what individual actors can achieve alone (2). Such consortia typically involve firms, universities and research institutes, and occasionally industry associations working jointly on pre-competitive or technical challenges, thereby forming complex innovation networks embedded in broader industrial systems.

TCM innovation consortia represent an organizational form centered on TCM enterprises, through which diverse innovation actors along the industrial chain are brought together to integrate complementary resources. By adopting a joint innovation model, these consortia aim to achieve technological breakthroughs while fostering an innovation ecosystem characterized by mutual dependence and co-evolution. In practice, innovation consortia have become an important strategic instrument for TCM enterprises to strengthen innovation capacity, share innovation risks, and enhance core competitiveness. Promoting industrial upgrading through the development of TCM innovation consortia is therefore of significance for advancing high-quality growth and improving international competitiveness, while also contributing to broader public health outcomes.

Member selection runs throughout both the formation and operational stages of innovation consortia. An appropriate member structure is crucial to their stable functioning. It not only enables the integration of complementary innovation resources, but also facilitates timely communication among members, thereby enhancing mutual trust, reducing unnecessary operational frictions, and improving overall efficiency and innovation returns. Prior research on innovation alliances and interorganizational networks demonstrates that member composition and network structure significantly affect collaborative performance and innovation outcomes (3, 4), with heterogeneous resource portfolios and trust-building interactions facilitating knowledge exchange and joint R&D effectiveness. However, the specific mechanisms driving member selection, especially in industry-specific consortia such as those in the TCM sector, remain underexplored.

This paper examines the key problems and influencing factors associated with member selection behavior in TCM innovation consortia. Based on this analysis, a set of hypotheses is proposed, followed by an evolutionary game analysis of member selection behavior. Drawing on the analytical results, the paper further puts forward policy recommendations for improving member selection mechanisms in TCM innovation consortia.

## 2 Literature review and theoretical foundations

Member selection constitutes a critical antecedent of collaborative innovation performance. In the context of TCM innovation consortia, the composition of members directly shapes resource integration, governance effectiveness, and long-term collaborative outcomes. Existing studies on member selection have gradually evolved from static, resource-oriented explanations toward more comprehensive frameworks that incorporate dynamic capabilities, relational governance, risk considerations, and network structures.

Early studies grounded in the resource-based view emphasize that complementarities among tangible and intangible resources provide the primary motivation for forming collaborative innovation arrangements (5). By partnering with organizations possessing differentiated technological, knowledge, or market resources, firms can compensate for internal limitations and achieve synergistic benefits. However, subsequent research increasingly questions whether static resource complementarity alone can sustain collaborative innovation over time.

Building on this critique, the dynamic capability perspective shifts attention from resource endowments to firms' abilities to sense, integrate, and reconfigure internal and external resources in response to environmental change (6). From a member selection standpoint, this implies that evaluating potential partners requires more than assessing existing assets; it also necessitates attention to their learning and adaptive capacities. Closely related is the concept of absorptive capacity, defined as a firm's ability to recognize, assimilate, and apply external knowledge (7). Empirical studies show that partners with stronger absorptive capacity are better positioned to integrate heterogeneous knowledge and enhance collaborative innovation performance (8). This consideration is particularly salient in TCM innovation consortia, where collaboration often spans experimental science, clinical practice, and experiential knowledge systems. Effective collaboration therefore depends not only on disciplinary complementarity but also on members' ability to translate and internalize knowledge across distinct epistemic domains.

Beyond resource and capability considerations, relational governance and social capital play a central role in member selection. Gulati (9) demonstrates that firms frequently form repeated alliances with prior partners, as trust, shared norms, and mutual understanding developed through past collaboration can significantly reduce transaction costs and cooperation risks. Uzzi (10) further distinguishes embedded ties from arm's-length market relations, arguing that embedded relationships—characterized by trust, fine-grained information exchange, and joint problem-solving—are especially conducive to the transfer of tacit knowledge. Given the tacit and experience-based nature of much TCM knowledge, trust-based embedded relationships are particularly valuable. Accordingly, assessments of partner credibility, reputation, and cultural compatibility constitute important, albeit informal, criteria in member selection.

At the same time, innovation consortia are inherently dynamic rather than static organizational forms. Lifecycle theories suggest that alliances typically move through stages of formation, operation, and potential restructuring, with different governance priorities emerging at each stage (11). From this perspective, member selection should be understood as the starting point of an ongoing adaptive process rather than a one-time decision. Extending this view, the notion of alliance portfolio reconfiguration highlights firms' need to continuously reassess and adjust their collaboration portfolios in response to shifting strategic objectives and technological environments (12). For TCM innovation consortia operating under changing regulatory conditions, evolving technological pathways, and uncertain market demand, selecting partners with strategic flexibility and long-term collaboration orientation becomes particularly important.

Member selection is also closely linked to the management of cooperation risks, especially opportunistic behavior. Transaction cost economics emphasizes that high asset specificity and environmental uncertainty increase the likelihood of opportunism in interorganizational collaboration (13). In innovation alliances, risks such as knowledge leakage, free riding, and unilateral learning followed by withdrawal are well documented (14). Consequently, evaluating potential partners requires attention not only to their resource contributions but also to their reputational history and propensity for opportunistic conduct. Governance mechanisms—such as clearly defined intellectual property arrangements and staged evaluation procedures—can partially mitigate these risks (15). In the TCM sector, where the allocation of rights over classical prescriptions and clinical data remains complex, clarifying expectations at the member selection stage is especially critical.

Finally, social network perspectives highlight that firms' positions within innovation networks affect their value as collaboration partners. Structural hole theory suggests that organizations bridging otherwise disconnected groups can access non-redundant information and brokerage advantages (16). Empirical evidence further indicates that such network positions can enhance innovation outcomes (17). In TCM innovation consortia, deliberately incorporating “bridging” organizations that connect research, clinical practice, industrial application, and raw material supply networks may help overcome information fragmentation and stimulate cross-domain innovation.

In sum, prior research provides a multidimensional understanding of member selection in innovation consortia, encompassing resources, capabilities, relational embeddedness, risk considerations, and network structures. However, much of this literature remains grounded in static assessments or ex post empirical correlations, offering limited insight into how member selection strategies evolve through repeated interaction under conditions of uncertainty and bounded rationality. This limitation is particularly pronounced in TCM innovation consortia, where knowledge heterogeneity and institutional complexity are high.

A substantial share of TCM knowledge is tacit in nature, embedded in practitioners' experience and organizational routines, and not easily captured through formal documentation. It includes clinical reasoning developed by experienced physicians, the intuitive assessment of medicinal material quality, know-how in processing and formulation, and adjustments of dosage based on patient responses. Unlike codified scientific knowledge, such tacit elements are typically learned through sustained interaction, joint problem solving, and repeated practice.

Because of this, collaboration in TCM innovation consortia cannot rely on one-off partner selection based on static resource evaluations. Instead, cooperation tends to unfold gradually, as partners interact over time, build mutual understanding, and adjust their expectations and behaviors through experience.

This also means that approaches based on static correlations are limited in explaining how member selection actually evolves. What is needed is a framework that can account for bounded rationality and learning under repeated interaction, where strategies are continuously adjusted based on experience and feedback. Evolutionary game theory offers such a perspective, as it focuses on how agents revise their strategies through imitation, learning, and payoff-driven adaptation. In this study, it is therefore used to analyze the dynamic process of member selection in TCM innovation consortia.

This pattern is not unique to China. Evidence from India indicates that conventional intellectual property systems often struggle to protect traditional medicine knowledge or to ensure fair benefit-sharing (18). This points to a broader mismatch between formal legal frameworks and indigenous knowledge systems, which is also relevant when considering how partners are selected and governed in TCM innovation consortia.

## 3 Determinants of member composition and selection in TCM innovation consortia

### 3.1 Partner composition of TCM innovation consortia

The primary objective of establishing TCM innovation consortia is to promote collaborative innovation and to facilitate the commercialization and industrialization of innovation outputs. In the process of joint innovation, consortium members capitalize on their respective comparative advantages, thereby maximizing individual benefits while sharing innovation-related risks. Such collaborative arrangements help to mitigate uncertainties associated with one-off transactions and to reduce the high risks that individual actors would otherwise bear independently. Scholars of strategic alliances and R&D consortia have similarly emphasized that pooling complementary resources across organizational boundaries is essential for joint innovation success, particularly in complex technological domains (19).

A well-developed TCM innovation consortium constitutes a complex innovation network comprising a diverse set of organizational actors. These include various types of TCM enterprises, universities, research institutes, intermediaries, financial institutions, and industry associations. Depending on differences in innovation strategies and resource endowments, consortium members assume distinct roles within the network. Among them, TCM enterprises, universities, and research institutes form the core of the consortium and are primarily responsible for innovation activities (20). Intermediaries, financial institutions, and industry associations constitute the peripheral layer, providing complementary and supportive functions for innovation (21). In multi-actor innovation networks, such peripheral actors often play critical roles in facilitating partner matching, bridging knowledge gaps, and reducing transaction costs — functions highlighted in both alliance network studies and innovation ecosystem research (22).

**Figure 1:**
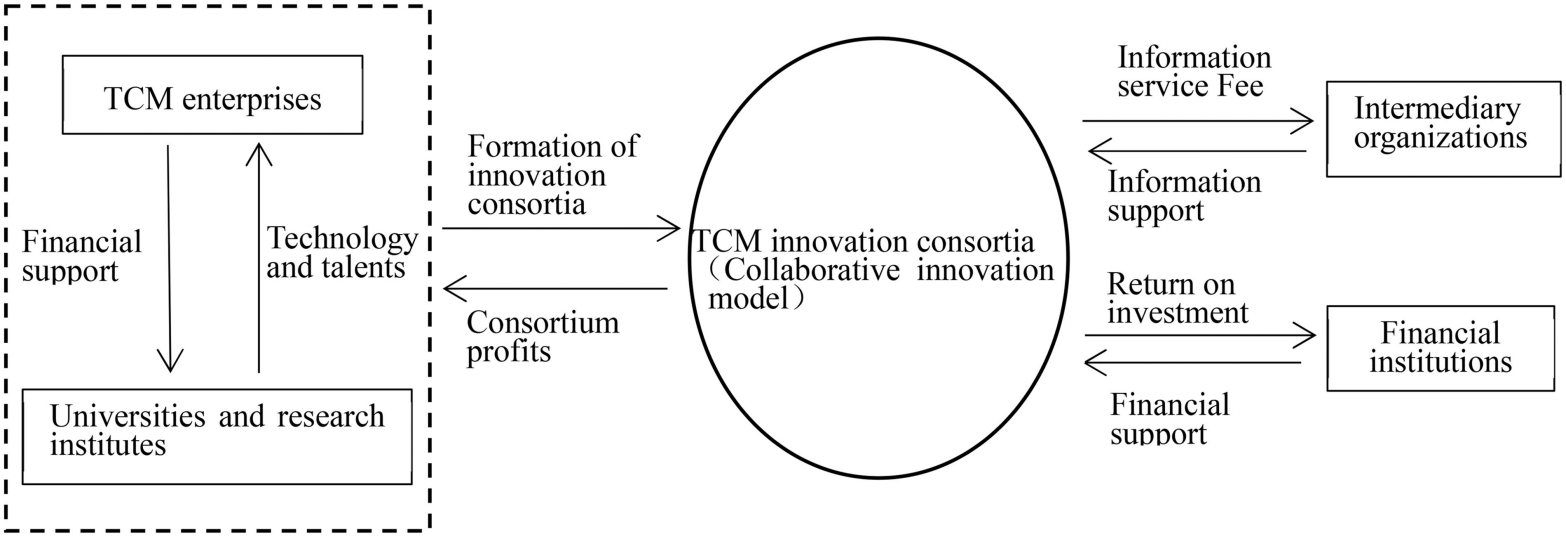
Members of the TCM innovation consortia.

The overall composition of members in a TCM innovation consortium is illustrated in Figure 1.

#### 3.1.1 TCM enterprises

TCM enterprises are the central actors in innovation consortia, distributed across upstream cultivation, midstream-processing, and downstream-distribution segments. Consortium-leading firms are typically processing enterprises producing decoction pieces or manufacturers of proprietary Chinese medicines. As market-oriented actors, these enterprises have direct exposure to end-user demand, enabling them to guide the direction of technological innovation. They also provide critical support to universities and research institutes, including R&D funding, access to facilities, and market-related information. Participation in collaborative innovation enhances firms' technological capabilities and leverages academic expertise and human capital. Ultimately enhancing competitiveness and promoting technological upgrading (23).

#### 3.1.2 Universities and research institutes

Universities and research institutes serve as primary sources of knowledge and technology, facilitating the translation of research into marketable innovation outcomes. Academic–research actors exchange intellectual resources for financial and practical support provided by TCM enterprises. Universities focus on basic and applied research, contributing to technological advancement and talent cultivation, while research institutes—whether government-led, independent, or enterprise-affiliated—emphasize applied research aligned with strategic innovation goals of consortium members (24).

#### 3.1.3 Intermediary organizations

Intermediaries, including chambers of commerce, consulting firms, and market research agencies, bridge knowledge gaps and facilitate communication both internally among consortium members and externally with government authorities. They reduce trial-and-error costs, enhance coordination, and contribute to a stable collaborative environment (25).

#### 3.1.4 Financial institutions

Banks, insurance companies, venture capital, and innovation funds provide essential financial support, influencing the stability and sustainability of consortia. Empirical evidence suggests that development financial institutions and other financial investors play a positive role in mobilizing finance and enhancing innovation activity (26).

Together, these actors form a cohesive innovation ecosystem in TCM consortia. Through resource complementarity and coordinated collaboration, they integrate dispersed capabilities, share innovation outcomes, and jointly pursue consortium objectives. Based on this analysis, the roles and interactions of these diverse actors provide a foundation for examining member selection mechanisms and optimizing collaborative innovation strategies in TCM consortia.

### 3.2 Factors affecting partner selection in TCM innovation consortia

The selection of members in TCM innovation consortia is influenced by multiple factors, including the complementarity of resources among innovation actors, transaction cost management capabilities, balance of overall strength, and the involvement of third-party forces. These factors not only determine the efficiency and stability of collaborative innovation but also directly affect the high-quality development of the TCM industry and the preservation of traditional medical knowledge.

#### 3.2.1 Complementarity of innovation resources

Resource complementarity among heterogeneous actors is the core driving force behind the formation of TCM innovation consortia. The TCM innovation chain covers the entire industry, from medicinal plant cultivation and raw material supply to decoction-piece processing, finished TCM production, and distribution channels. Different innovation actors naturally possess heterogeneous resources, and no single actor can cover the full chain from basic research, technology development, to commercialization on its own. When critical innovation resources cannot be efficiently acquired through the market, or internal development is too costly or time-consuming, forming a consortium to share resources and complement strengths becomes the optimal solution.

The core innovation resources of a TCM consortium include funding, technical capability, human capital, and market channels. The degree of complementarity among these resources directly determines the consortium's innovation potential and operational performance. TCM enterprises, due to their position in the mid- and downstream segments of the industry chain, possess market sensitivity, mature sales channels, and brand influence, making them essential for the commercialization and practical application of innovations.

The degree of resource complementarity therefore constitutes a major source of cooperative surplus and provides the basis for the payoff structure adopted in the subsequent evolutionary game analysis (27).

##### 3.2.1.1 Funding

Funding constitutes the cornerstone of innovation activities within TCM innovation consortia. In practice, TCM enterprises typically provide the primary source of R&D funding through internal financial resources, while financial institutions offer supplementary support in the form of credit, investment, and risk-sharing mechanisms. The scale and stability of funding directly affect firms' capacity to sustain long-term R&D activities and to transform technological outputs into commercially viable products.

In the context of China's TCM industry—characterized by low industrial concentration and the predominance of small- and medium-sized enterprises—resource constraints are particularly salient. Limited internal financing capacity increases firms' reliance on collaborative arrangements to pool resources and mitigate innovation risks. Consequently, potential partners' funding capacity serves not only as an indicator of innovation commitment, but also as a signal of their ability to endure uncertainty and maintain long-term cooperation.

Accordingly, funding capacity is reflected in the model as a key determinant of both cooperation costs and expected returns, consistent with prior research indicating that financial resources significantly influence partner selection and expected benefits in R&D collaborations (28).

##### 3.2.1.2 Technology and talent

Universities and research institutes serve as core providers of technical expertise and talent. TCM enterprises, positioned at the core of the industry chain, are responsible for translating academic outcomes into commercially viable products. By collaborating with academic actors, TCM enterprises gain access to patents, attract skilled personnel, and continuously receive innovation support, creating a dual engine of technology and market capability. Assessment of academic actors' innovation capacity should include metrics such as the number and quality of intellectual property rights, coverage of core technologies, and efficiency in translating innovations into results. Such collaboration optimizes the R&D chain, ensures the advanced nature of products, and strengthens the consortium's overall innovation quality.

These technological and human-capital advantages are incorporated into the model as factors affecting the marginal returns of cooperative strategies.

##### 3.2.1.3 Market channels

Market channels are crucial for commercializing innovation outcomes. TCM enterprises leverage established sales networks, brand recognition, and market penetration to transform innovations into products and deliver them to end-users efficiently. Intermediary organizations provide professional matchmaking and consulting services, ensuring the smooth commercialization of technological achievements. Therefore, market coverage, commercialization experience, and brand influence are critical criteria in partner selection.

Market-related advantages thus influence the probability that cooperative innovation yields positive payoffs.

#### 3.2.2 Transaction cost management

Transaction costs in a TCM consortium include negotiation, monitoring, and risk mitigation costs. The long-term stability of the consortium depends on effective control of these costs, as highlighted in research on strategic alliances emphasizing the role of transaction costs in alliance efficiency and sustainability (29). The TCM industry is characterized by complex supply chains and long R&D cycles, making cost control essential for efficient collaboration.

##### 3.2.2.1 Search and negotiation costs

Search costs arise from information asymmetry, as TCM enterprises evaluate the technical capability and potential of academic partners, while academic actors assess the financial stability, market channels, and reputation of enterprises. Intermediary and financial organizations perform due diligence to ensure investment safety. Negotiation costs are higher than one-off market transactions due to the specificity of innovation resources (e.g., proprietary TCM formulas, core technologies) and the long-term nature of collaboration. Multi-round negotiations cover contract duration, benefit allocation, and risk-sharing. Alignment of strategic goals and interests is key to controlling negotiation costs.

Such costs are treated as initial cooperation costs that influence strategic choice under bounded rationality.

##### 3.2.2.2 Monitoring and compliance costs

TCM consortia function as loosely structured innovation networks that require monitoring mechanisms to ensure timely delivery of technology and commercialization outcomes. Monitoring costs include system design, performance verification, and breach management, while compliance costs involve preventive measures against risks such as delayed delivery, insufficient funding, or information distortion. Selecting partners with strong risk resilience and reliable performance records is critical for reducing costs and ensuring stable operations.

Monitoring and compliance costs are therefore reflected in the dynamic adjustment process of strategies in the evolutionary game.

#### 3.2.3 Balance of overall strength

The balance of overall strength among consortium members is essential for long-term stability. TCM enterprises, as core actors in the industry chain, hold significant influence within the consortium. Balanced member composition prevents dominance by any single actor, reduces free-riding behavior, and ensures smooth progress of collaborative innovation. Partner selection should prioritize comparable or controllable differences in strength to build a stable, complementary consortium.

Relative strength balance is thus introduced as a key parameter shaping the evolutionary stability of cooperative outcomes.

#### 3.2.4 Third-party forces

Third-party actors play an important role in providing information and coordinating governance in member selection. These include government agencies, industry associations, and technology intermediaries. Governments can guide and supervise consortium activities through policy, financial support, legal oversight, and credit system development, ensuring stable operation. Non-governmental organizations provide consultation, coordination, and technology transfer services, reducing hidden costs such as time and adjustment in partner selection and early collaboration. While third-party involvement does not change the inherent resources of consortium members, it provides accurate information, lowers search costs, and enforces penalties for opportunistic behaviors, thereby promoting stable and effective collaboration. Governments receive indirect benefits, while industry associations and technology intermediaries can participate in the allocation of innovation outcomes and obtain direct returns.

These mechanisms provide the institutional foundation for introducing third-party-related cost, fee, and penalty parameters in the subsequent evolutionary game model.

## 4 Evolutionary game analysis of member selection in TCM innovation consortia

In TCM innovation consortia, various innovation actors engage in collaborative innovation to share risks, resources, and benefits. The decision-making process of core enterprises' collaboration decisions (19). Stable strategies in this evolutionary game can be identified through evolutionary equilibrium analysis and replicator dynamics, while resource allocation issues during collaboration can also be explored within this framework (30). As consortium members accumulate experience and time progresses, the ecosystem continuously adjusts through trial-and-error learning, gradually approaching an optimized state.

Previously, member behavior was described in terms of participation and non-participation, indicating whether actors join the consortium. In the evolutionary game model, we focus on strategic interactions, represented by cooperation and defection, while the broader notion of participation remains relevant outside the game-theoretic context.

### 4.1 Mathematical analysis of evolutionary game

Evolution represents a dynamic process of “survival of the fittest,” and evolutionary game models focus on group decision-making (31). Under the assumption of bounded rationality, any player in the game may adopt a range of strategies. Optimal strategies do not emerge immediately; they evolve progressively through repeated interactions. The formation of strategy equilibria is thus a dynamic process, and equilibrium states are dynamic and may change over time (32). Since the 1980s, scholars have applied evolutionary game theory and replicator dynamics to study the stability of strategies in organizational and technological contexts, demonstrating its broad applicability to innovation alliances and R&D consortia.

Replicator dynamics is a mechanism for the dynamic adjustment of strategies within a population. It reflects the tendency of individuals to increasingly adopt strategies that have proven more successful in repeated, randomly paired interactions. Formally, the frequency of a strategy being adopted is represented by a differential equation:

$$\frac{dx_{k}}{dt}\;=\;x_{k}[u(k,\;s)\;-u(s,\;s)]$$ (1)

Where *k* denotes a specific strategy, *x_k_* is the proportion of the population adopting strategy *k* at time *t, u*(*k, s*) is the expected payoff of strategy *k*, and *u*(*s*, *s*) is the average payoff of the population. When the payoff of a strategy exceeds the population average—i.e., $\frac{dx_{k}}{dt}/x_{k}\;>\;0$ —the strategy propagates, increasing the number of individuals choosing it. Conversely, if its payoff is below the population average, adoption declines.

In bounded rationality games, the focus is not on optimal strategy selection *per se*, but on the dynamic adjustment, evolutionary trends, and stability of strategies. The Evolutionarily Stable Strategy (ESS) is central in evolutionary game theory. It is a strategy that, if adopted by most individuals, cannot be invaded by a small number of mutants.

Formally, a strategy *s* is an evolutionarily stable strategy (ESS) if, for any alternative strategy k≠s, either *u*(*s*, *s*) > *u*(*k, s*), or *u*(*s*, *s*) = *u*(*k, s*) and *u*(*s, k*) > *u*(*k, k*).

Any stable point of an evolutionary game is necessarily a Nash equilibrium, although not all Nash equilibria are evolutionarily stable. Under replicator dynamics, an ESS is guaranteed to be an evolutionary equilibrium. To illustrate, consider a general two-player symmetric game with two strategies—cooperation (Strategy 1) and defection (Strategy 2)—with the corresponding payoff matrix shown in Table 1.

**Table 1:** Payoff matrix for a symmetric two-player game.

		Player2	
		Cooperation	Defection
Player1	Cooperation	*a, a*	*b, c*
	Defection	*c, b*	*d, d*

Let *x* denote the proportion of the population adopting cooperation, and (1−x) the proportion adopting defection. The expected payoffs of cooperation and defection, and the average payoff of the population, are given by:“ to: ”The expected payoffs of cooperation and defection are given by Equations (2) and (3), and the average payoff of the population by Equation (4).

$$u_{1}\;=\;ax-b(1-x)$$ (2)

$$u_{2}\;=\;cx\;+\;d(1-x)$$ (3)

$$\bar{u}\;=\;u_{1}x\;+\;u_{2}(1-x)$$ (4)

Based on Equation (1), the replicator dynamic equation for cooperation is given by:“ to: ”the replicator dynamic equation for cooperation is given by Equation (5):

$$F(x)\;=\;\frac{dx}{dt}\;=\;x(u_{1\;}-\bar{u})\;=\;x(1-x)[(a\;+\;d-b-c)x\;+\;b-d]$$ (5)

**Figure 2:**
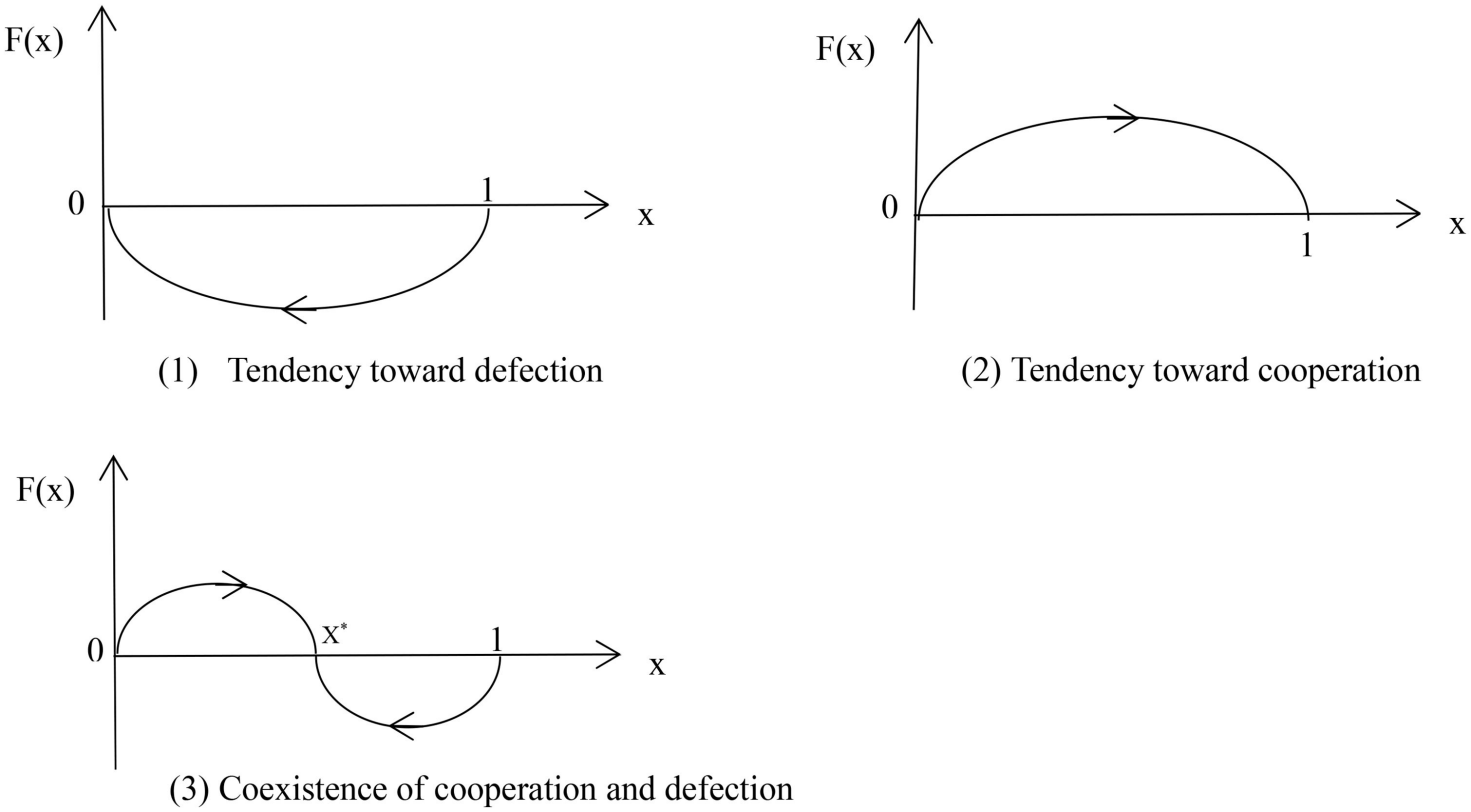
Evolutionary phase diagram of joint strategies.

Setting F(x)=0 yields three fixed points of the replicator dynamic equation, namely:

$x^{*}=0,\;\;x^{*}\;=\;1,\;\;x^{*}\;=\;\frac{d-b}{a+d-b-c}$, The phase diagram of this equation is shown in Figure 2.

The ESS can be identified from the phase diagram, as summarized in Table 2.

**Table 2:** Evolutionarily stable strategies for pairwise symmetric games.

Conditions	Evolutionarily stable strategies	Stable outcomes
$\frac{d-b}{a\;+\;d-b-c}\;<\;0$	X* = 0	Defection
$\frac{d-b}{a\;+\;d-b-c}\;>\;1$	X* = 1	Cooperation
$0\;<\;\frac{d-b}{a\;+\;d-b-c}\;<\;1$	$x^{*}\;=\;\frac{d-b}{a\;+\;d-b-c}$	Coexistence

Specifically:

When $\frac{d-b}{a\;+\;d-b-c}\;<\;0$, all individuals adopt defection. Even if a small fraction initially participates, the population gradually shifts toward defection, ultimately reaching an evolutionarily stable state.

When $\frac{d-b}{a\;+\;d-b-c}\;>\;1$, all individuals adopt cooperation. Even if some initially choose defection, the population eventually converges to full cooperation.

When $0\;<\;\frac{d-\;b}{a\;+\;d-b-c}\;<\;1$, the system reaches an interior equilibrium $x^{*}=\frac{d-b}{a+d-b-c}$, representing a coexistence of cooperation and defection. If the proportion of cooperators exceeds x*, defection becomes relatively more advantageous, and some individuals switch to defection until the proportion returns to x*. Conversely, if the proportion of cooperators falls below x*, cooperation becomes more advantageous, and the number of participants rises until the system stabilizes at x*. The phase diagram and ESS analysis provide a theoretical foundation for modeling strategic decisions in TCM consortia and serve as the basis for subsequent simulation studies.

### 4.2 Evolutionary game analysis of collaborative behavior in TCM consortia

A TCM consortium can be regarded as an ecosystem composed of multiple innovative actors, with each actor representing a distinct “species.” Within this ecosystem, the competitive and cooperative relationships among these species form the network structure of the entire system. Research on collaborative innovation within TCM consortia primarily focuses on the internal innovation activities of the consortium, specifically on how core enterprises select suitable partners and implement innovation strategies under the constraints of consortium contracts.

#### 4.2.1 Bounded rationality and strategic evolution in TCM consortia

Collaborative innovation within TCM consortia is a dynamic, multi-actor process involving core enterprises—primarily TCM pharmaceutical companies—as well as universities, research institutes, intermediaries, and clinical partners. TCM R&D exhibits unique characteristics, including complex multi-component formulas, variability in raw herbal materials, traditional production processes, and extended clinical validation cycles. These factors, coupled with regulatory uncertainties and fluctuating market conditions, constrain the ability of actors to fully anticipate all potential scenarios, making it impossible to predetermine optimal collaboration strategies.

Consequently, member selection and strategy optimization in TCM consortia are inherently iterative. Core enterprises gradually adjust their partnerships, formulation designs, and collaborative approaches based on experimental outcomes, accumulated experience, and market feedback. For instance, a TCM enterprise may favor partners who demonstrate higher R&D efficiency, strict adherence to quality standards, or reliable sourcing of medicinal materials, while reducing collaboration with less effective partners. This adaptive learning process reflects the bounded rationality of consortium actors and aligns naturally with the principles of evolutionary game theory, particularly replicator dynamics, where the frequency of successful strategies increases over time.

In this context, a TCM innovation consortium can be conceptualized as an evolving ecosystem of interdependent actors, whose interactions, trial-and-error learning, and resource integration collectively drive technological innovation. The bounded rationality of members not only explains the inherent incompleteness of consortium contracts but also justifies the use of evolutionary game models to analyze how TCM enterprises strategically select partners and allocate resources within such consortia.

#### 4.2.2 Optimization of collaborative innovation strategies in TCM innovation consortia

The process of collaborative innovation within TCM innovation consortia is characterized by dynamic learning and resource integration between core enterprises and their partners. Core enterprises may simultaneously establish contractual collaborative relationships with multiple innovation actors, thereby integrating complementary resources across different entities to enhance overall innovation efficiency. Owing to bounded rationality, however, core enterprises are often unable to accurately determine ex ante which innovation partners are most advantageous for collaboration. Instead, they adjust and optimize their partner selection strategies dynamically based on accumulated experience and available information. Such adaptive optimization processes correspond closely to the replicator dynamics framework in evolutionary game theory.

In this regard, member selection in TCM innovation consortia constitutes a gradual process of learning and evolution. A TCM innovation consortium can thus be conceptualized as an evolutionary ecosystem composed of multiple boundedly rational innovation actors, rendering evolutionary game theory an appropriate analytical framework for examining collaborative innovation behavior within such consortia.

### 4.3 Basic hypotheses of the evolutionary game model

Member selection in a TCM consortium is a process involving interactions among multiple actors. The consortium includes various innovative entities such as TCM manufacturing enterprises, universities, research institutes, intermediary organizations, and financial institutions, each playing distinct roles within the consortium. Among these, TCM enterprises and academic-research institutions form the core layer of the consortium, bearing the primary responsibility for innovation and R&D; intermediaries, financial institutions, and similar actors constitute the peripheral support layer, providing auxiliary functions to facilitate collaborative innovation.

Decisions on member selection are based on whether to cooperate: choosing “cooperation” establishes a collaborative relationship, allowing the consortium to be formed and operate; choosing “defection” represents the termination of the relationship, preventing the consortium from forming and potentially leading to dissolution, thereby undermining its stability.

Based on the foregoing analysis of factors influencing member selection in TCM consortia, the following hypotheses are proposed:

Hypotheses 1: The innovative actors participating in the member-selection game of a TCM consortium are limited to two parties: the leader (TCM enterprise) and the partners (universities and research institutes). As the backbone of the consortium's innovation activities, these two parties are the backbone of the consortium and play a decisive role in its stable operation. In the game, the leader and the partners are considered equal in status, and their strategy choices are independent of whether they take an active or passive role during the consortium's formation.

Hypotheses 2: Both parties in the game exhibit bounded rationality. That is, the decision-making behavior of each innovative actor is not fully rational but constrained by factors such as cognitive capabilities and external environments. Bounded rationality implies that member selection is not a one-time decision; rather, it is a process in which the leader and the partners gradually reach consensus through continuous learning, trial-and-error, and experience accumulation. Although differences in preferences may exist, the ultimate goal of both parties is to maximize their own interests.

Hypotheses 3: Member selection occurs through random matching. The innovative actors participating in the formation of the consortium are not predetermined; cooperation between the leader and the partners is established via randomly selected pairings. The selection game is repeatedly played between core enterprises and potential partners until both parties reach a mutual agreement to collaborate.

Hypotheses 4: The member-selection game belongs to the category of non-zero-sum games. Participation of innovative actors in a TCM consortium can lead to a multi-party win-win outcome, where the magnitude of each actor's payoff does not depend on the number of members, but rather on their own resource contributions and those of other members. Let the returns on resource investment for the leader and the partners be denoted by *α* and *β*, respectively, with 0 < *α* < 1, 0 < *β* < 1. The values of *α* and *β* are primarily influenced by the complexity of the innovation technology and market uncertainty: smaller values of *α* and *β* indicate higher technological complexity and greater market uncertainty, whereas larger values indicate simpler technology and lower market uncertainty.

Hypotheses 5: The strategy options for both parties in the game are limited to “cooperation” and “defection,” with each party's strategy set given by *S* *=* {s_1_, s_2_}. Where s_1_ = cooperation and s_2_ = defection. Let the probability that the leader chooses strategy s_1_ be x (0 ≤ x ≤ 1), and the probability of choosing s_2_ be 1−*x*; likewise, let the probability that the collaborative partner chooses s_1_ be y (0 ≤ y ≤ 1) and the probability of choosing s_2_ be 1−*y*.

Hypotheses 6: Let C_1_, C_2_ denote the total costs incurred by the leader and the collaborative partner, respectively, in participating in the formation of a TCM consortium. These costs include both resource investment costs and various types of transaction costs.

Hypotheses 7: Let *λ* and *θ* represent the resource complementarity coefficient and the relative comprehensive strength parameter between the leader and the collaborative partner, respectively, with 0 < *λ* < 1 and 0 < *θ* < 1. A value of *λ* closer to 0 indicates weaker resource complementarity, corresponding to higher coordination costs; even if cooperation is achieved, one party will find it difficult to gain substantial benefits from the other party's resource contributions. The benefits that the leader and the collaborative partner obtain from resource complementarity are assumed to be *λ*C_2_ and *λ*C_1_, respectively.

A value of *θ* closer to 0 indicates a larger gap in comprehensive strength between the two parties, whereas a value closer to 1 indicates more balanced comprehensive strength. The benefits that the leader and the collaborative partner obtain based on their relative comprehensive strength are assumed to be *θ*C_2_ and *θ*C_1_, respectively.

Hypotheses 8: If both parties choose the “defection” strategy, meaning that neither joins the TCM consortium, the payoffs for the leader and the collaborative partner are denoted as u_1_ and u_2_, respectively.

Hypotheses 9: The cost savings in member selection within a TCM consortium resulting from the involvement of a third party are denoted by C_s_, with C_s1_ representing the cost saved by the leader and C_s2_ representing the cost saved by the collaborative partner.

Hypotheses 10: After providing support for member selection in a TCM consortium, the third party receives a benefit return denoted by C_r_ (for simplicity, any implicit benefits gained by a governmental third party are also converted into direct benefit returns). This cost is shared equally by the leader and the collaborative partner, meaning that both parties are required to pay a service fee of C_r_.

Hypotheses 11: The third party plays a coordinating and supervisory role in the member-selection process of a TCM consortium and imposes penalties on any party that breaches the agreement. Let the breach cost be *R*, which is awarded as compensation to the party whose interests are harmed. If both parties fail to fulfill their obligations, the consortium cannot be formed, and no breach penalty is imposed.

The relevant parameters and their definitions are presented in Table 3.

**Table 3:** Model parameters and their definitions.

Parameters	Definitions
*α*	The rate of return on the leader's resource investment, 0 < *α* < 1
*β*	The rate of return on the collaborative partner's resource investment, 0 < *β* < 1
C_1_	The total cost incurred by the leader in participating in the formation of the TCM consortium, including resource investment costs and various transaction costs
C_2_	The total cost incurred by the collaborative partner in participating in the formation of the TCM consortium, including resource investment costs and various transaction costs
*λ*	The resource complementarity coefficient between the leader and the collaborative partner, 0 < *λ* < 1
*θ*	The relative overall strength parameter between the leader and the collaborative partner, 0 < *θ* < 1
U_1_	The payoff of the leader when both parties choose the “defection” strategy
U_2_	The payoff of the collaborative partner when both parties choose the “defection” strategy
C_s_	The cost savings in member selection due to the involvement of a third party
C_s1_	The cost savings for the leader due to third-party involvement
C_s2_	The cost savings for the collaborative partner due to third-party intervention
C_r_	The benefit or return obtained by the third party
R	The penalty for breaching the agreement

Based on the above assumptions, the payoff matrix for the two parties in the TCM consortium game can be obtained, as shown in Table 4:

**Table 4:** Payoff matrix of the TCM consortium game.

Strategy		Partner Strategy	
		Cooperation *y*	Defection 1-y
Leader	Cooperation x	$u_{1}\;+\;\alpha C_{1}\;+\;\lambda \theta C_{2}-C_{1}\;+\;C_{s1}-C_{r}$ $u_{2}\;+\;\beta C_{2}\;+\;\lambda \theta C_{1}-C_{2}\;+\;C_{s2}-C_{r}$	$u_{1}\;+\;\alpha C_{1}-C_{1}\;+\;C_{s1}-C_{r}\;+\;R$ $u_{2}-R$.
	Defection 1−x	$u_{1}-R$ $u_{2}\;+\;\beta C_{2}-C_{2}\;+\;C_{s2}-C_{r}\;+\;R$	u_1_ u_2_

### 4.4 Stability and dynamic evolution of the member-selection game in TCM consortia

Let the expected payoffs for the leader in the TCM consortium when choosing the “cooperation” and “defection” strategies be $u_{1}^{s}$ and ${\;u}_{1}^{r}$, respectively, and let the average expected payoff be $\;\;{\bar{u}}_{1}$. Based on the payoff matrix, we have:“ to: ”Based on the payoff matrix, we have the expected payoffs as expressed in Equations (6–8):

$$\;\;\;\;u_{1}^{s}\;=\;y(u_{1}\;+\;\alpha C_{1}\;+\;\lambda \theta C_{2}-C_{1}\;+\;C_{s1}-C_{r})\;+\;(1-y)(u_{1}\;+\;\alpha C_{1}-C_{1}\;+\;C_{s1}-C_{r}\;+\;R\;)$$

$$=u_{1}\;+\;(\alpha -1)C_{1}\;+\;y(\lambda \theta C_{2}-R)\;+\;C_{s1}-C_{r}\;+\;R$$ (6)

$$u_{1}^{r}\;=\;y(u_{1}-R)\;+\;(1-y)u_{1}\;=\;u_{1}-\mathrm{yR}$$ (7)

$${\bar{u}}_{1}={\mathrm{xu}}_{1}^{s}\;+\;(1-x)u_{1}^{r}$$ (8)

The replicator dynamic equation for the core enterprise joining the TCM consortium is given by:

$$F(x)\;=\;\frac{\mathrm{dx}}{\mathrm{dt}}\;=\;x(u_{1}^{s}-{\bar{u}}_{1})\;=\;x\;(1-x)[(\alpha \;-1)C_{1}\;+\;y\lambda \theta C_{2}\;+\;C_{s1}-C_{r}\;+\;R]$$ (9)

Setting F(x) = 0 three stationary conditions, namely: $y^{*}\;=\;\frac{(1-\alpha )C_{1}\;+\;C_{r}-C_{s1}-R}{\lambda \theta C_{2}}$, $x_{1}^{*}\;=\;0,\;x_{2}^{*}\;=\;1$. Let the expected payoffs for the collaborative partner in the TCM consortium when choosing the “cooperation” and “defection” strategies be $u_{2}^{s}$ and $u_{2}^{r}$, respectively, and let the average expected payoff be $\;{\bar{u}}_{2}$. Similarly, the replicator dynamic equation for the collaborative partner joining the TCM consortium can be obtained as:

$$F(y)\;=\;\frac{\mathrm{dy}}{\mathrm{dt}}\;=\;y(u_{2}^{s}-{\bar{u}}_{2})\;=\;y(1-y)[(\beta -\;1)C_{2}\;+\;x\lambda \theta C_{1}\;+\;C_{s2}-C_{r}\;+\;R\;]$$ (10)

Setting F(y) = 0 yields three equilibrium points, namely: $x^{*}\;=\;\frac{(1-\beta )C_{2}\;+\;C_{r\;}-C_{s2}-R\;}{\lambda \theta C_{1}}$,$\;y_{1}^{*}\;=\;0,\;y_{2}^{*}\;=\;1$. From the above calculations, five equilibrium points can be identified in the plane S={(x,y)|0≤x≤1,0≤y≤1}, namely: (0, 0), (0, 1), (1, 0), (1, 1)和(x*, y*). The first four points are pure strategy equilibria, while (x*, y*) is a saddle point, representing a mixed strategy equilibrium.

Differential equations (9) and (10) constitute the replicator dynamic system for the member-selection game in a TCM consortium, with their equilibrium points forming the evolutionary game equilibria.

### 4.5 Dynamic evolution of the member-selection game in TCM consortia

By taking the partial derivatives of differential equations (9) and (10) with respect to *x* and *y*, the Jacobian matrix of the evolutionary game system for the TCM consortium can be obtained. The stability of the five equilibrium points is then determined through the local stability analysis of this matrix.

The Jacobian matrix is as follows in Equation (11), with its determinant in Equation (12), trace in Equation (13):

$$\;J=\left[\begin{array}{cc} \begin{array}{c} \left(1-2x)[(\alpha -1)C_{1}\;+\;y\lambda \theta C_{2}\;+\;C_{s1}-C_{r}\;+\;R\;\right]\\ y(1-y)\lambda \theta C_{1} \end{array} & \begin{array}{c} x(1-x)\lambda \theta C_{2}\\ \left(1-2y)[(\beta -1)C_{2}\;+\;x\lambda \theta C_{1}\;+\;C_{s2}-C_{r}\;+\;R\;\right] \end{array} \end{array}\right]$$ (11)

The determinant of the Jacobian matrix, det(*J*), is:

$$\frac{\partial F(x)}{\partial x}\;\times \;\frac{\partial F(y)}{\partial y}-\frac{\partial F(x)}{\partial y}\;\times \;\frac{\partial F(y)}{\partial x}$$ (12)

The trace of the Jacobian matrix, tr(*J*), is:

$$\frac{\partial F(x)}{\partial x}\;+\;\frac{\partial F(y)}{\partial y}$$ (13)

The stability properties of the equilibrium points are examined using the local stability analysis method proposed by Friedman (1991). If det(*J*) > 0 and tr(*J*) *<* 0, the corresponding equilibrium point is asymptotically stable; if det(*J*) > 0 and tr(*J*) > 0, the equilibrium point is unstable; and if det(*J*) *<* 0, the equilibrium point is a saddle point.

By substituting the five equilibrium points (0, 0), (0, 1), (1, 0), (1, 1) and (x*, y*) into the Jacobian matrix, the resulting determinants and traces are shown in Table 5.

**Table 5:** Local stability of equilibria.

Equilibrium	det(*J*)	tr(*J*)	Local stability
0, 0	+	−	ESS
0, 1	+	+	Unstable
1, 0	+	+	Unstable
1, 1	+	−	ESS
x*, y*	−	0	Saddle point

The saddle point is given by Equation (14):

$$(x^{*},\;y^{*})\;=\;(\frac{(1-\beta )C_{2}\;+\;C_{r}-C_{s2}-R}{\lambda \theta C_{1}},\;\frac{(1-\alpha )C_{1}\;+\;C_{r}-C_{s1}-R}{\lambda \theta C_{2}})$$ (14)

**Figure 3:**
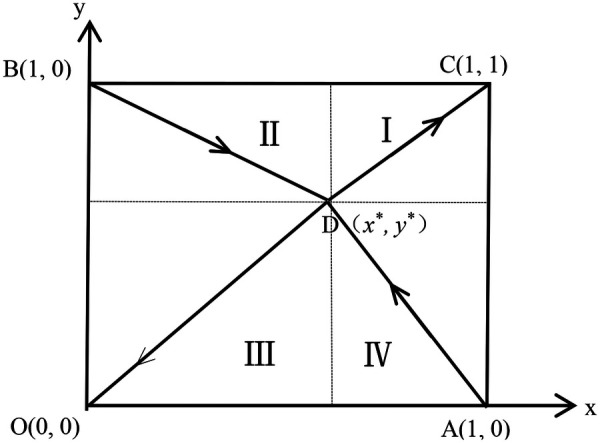
Evolutionary phase diagram of equilibria for TCM consortium members.

Based on the local stability analysis in Table 5, the evolutionary phase diagram of the equilibrium points can be obtained, as shown in Figure 3.

As shown in Figure 3, the phase diagram is divided into four regions by a dashed line passing through the saddle point D, with evolution within each region primarily occurring along the curves DC, BD, DO and AD. When the initial state of the TCM consortium game (x_0_, y_0_) falls within Region I, both parties have strong willingness to cooperate, allowing the consortium to be formed and carry out innovation activities, ultimately evolving toward the state (1, 1). In this case, the TCM consortium is successfully established, or existing member relationships are further consolidated, enhancing the consortium's stability. Even if the cooperative relationship between the two parties undergoes some changes or is disturbed by internal and external factors, the impact on the stable state of the consortium remains limited.

Conversely, when the initial state (x_0_, y_0_) lies within Region III, both parties exhibit low willingness to cooperate. Under these conditions, the TCM consortium cannot be formed, or existing partnerships gradually dissolve, leading to a decline in consortium stability and eventual evolution toward (0, 0).

When the initial state of the TCM consortium game (x_0_, y_0_) falls within Region II, the leader has a strong willingness to join the consortium, while the collaborative partner may adopt certain reasonable strategies to increase the likelihood of cooperation. As the number of interactions between the two parties increases, if the joint returns fail to meet the collaborative partner's prior expectations, their probability of joining the consortium decreases to y*, while the leader's probability of joining gradually rises to x*. Consequently, the TCM consortium gradually evolves toward the saddle point D. Although D represents an equilibrium state, it is not a stable point of the system, and even minor disturbances can alter this equilibrium.

Similarly, when the initial state (x_0_, y_0_) lies within Region IV, the TCM consortium evolves along the AD direction toward the saddle point D. Upon reaching D, the system attains an equilibrium state; however, this equilibrium is likewise not stable.

Within the four regions described above, the area of Region I is positively correlated with the probability that the system will evolve to the stable state (1, 1), while the area of Region III is positively correlated with the probability of evolving to the stable state (0, 0). For Regions II and IV, even minor disturbances within these regions can affect the system's eventual stable state.

**Figure 4:**
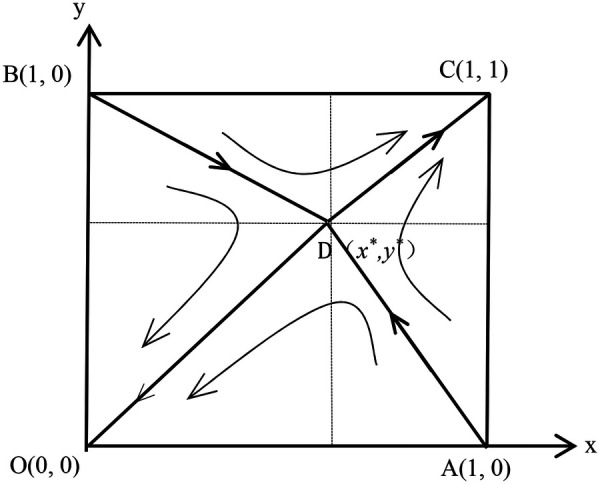
Evolutionary dynamics diagram of TCM consortium members’ strategy selection.

Therefore, by dividing the phase diagram along the polyline ADB into two parts, disturbances occurring in the S_ADBC_ region ultimately evolve toward the stable state (1, 1), whereas disturbances in the S_ADBO_ region ultimately evolve toward (0, 0). Based on this, the dynamic evolution diagram of member selection in the TCM consortium can be constructed, as illustrated in Figure 4.

As shown in Figure 4, the stable strategies corresponding to member selection in the TCM consortium are (cooperation, cooperation) and (defection, defection). Assuming that the initial states of the TCM consortium members are uniformly and randomly distributed within the region *S* = [0, 1] × [0, 1], and using the polyline ADB as a boundary, the evolutionary trend of member selection and the resulting state of the consortium can be determined by analyzing the relative areas of S_ADBC_ and S_ADBO_. If the expected final evolutionary outcome of the consortium is (cooperation, cooperation), the condition that must be satisfied is S_ADBC_ *>* S_ADBO_.

As shown in Figure 4, the area of region ADBC is:

$$S_{\mathrm{ADBC}}\;=\;1-\frac{1}{2}(x^{*}\;+\;y^{*})=1-\frac{\left(1-\beta )C_{2}^{2}\;+\;(1-\alpha )C_{1}^{2}\;+\;(C_{1}\;+\;C_{2})(C_{r}-R)-(C_{1}C_{s1\;}+\;C_{2}C_{s2}\right)}{2\lambda \theta C_{1}C_{2}}$$ (15)

When $0\;<\;x^{*}\;+\;y^{*\;}<\;1$, that is, $0\;<\;\frac{\left(1-\beta )C_{2}^{2}\;+\;(1-\alpha )C_{1}^{2}\;+\;(C_{1}\;+\;C_{2})(C_{r}-R)-(C_{1}C_{s1\;}+\;C_{2}C_{s2}\right)}{\lambda \theta C_{1}C_{2}}\;<\;1$, we have $S_{\mathrm{ADBC}}\;>\;S_{\mathrm{ADBO}}$, and the evolutionary stable strategy tends toward (cooperation, cooperation).

When $x^{*}\;+\;y^{*}\;=\;1$, analogously, when we have S_ADBC_ = S_ADBO_, and the probabilities of evolving to (cooperation, cooperation) and (defection, defection) are equal. When $1\;<\;x^{*}\;+\;y^{*}\;<\;2$, we have S_ADBC_ *<* S_ADBO_, and the evolutionary stable strategy tends toward (defection, defection).

### 4.6 Main findings from the evolutionary game analysis

#### 4.6.1 Corollary 1

Changes in the costs incurred by both parties in the game have a significant impact on whether the consortium is formed.

By taking the partial derivatives of Equation (15) with respect to C_1_ and C_2_, we obtain Equations (16) and (17):

$$\frac{\partial S_{\mathrm{ADBC}}}{\partial C_{1}}\;=\;\frac{(1-\alpha )C_{1}^{2\;}+(C_{r}-R-C_{s1})C_{1}+(1-\beta )C_{2}^{2}+C_{2}(C_{r}-R-C_{s2})-2(1-\alpha )C_{1}C_{2}-(C_{r}-R-C_{s1})C_{2}}{2\lambda \theta C_{1}^{2}C_{2}}$$ (16)

$$\frac{\partial S_{\mathrm{ADBC}}}{\partial C_{2}}\;=\;\frac{(1-\beta )C_{2}^{2}+(C_{r}-R-C_{s2})C_{2}+(1-\alpha )C_{1}^{2}+C_{1}(C_{r}-R-C_{s1})-2(1-\beta )C_{1}C_{2}-(C_{r}-R-C_{s2})C_{1}}{2\lambda \theta C_{1}C_{2}^{2}}$$ (17)

It can be observed that both the numerator and denominator contain higher-order terms, so the derivatives are non-negligible functions of C_1_ and C_2_. Therefore, the marginal effects of C_1_ and C_2_ on S_ADBC_ are significant, indicating that changes in the costs incurred by both parties have a substantial impact on the convergence outcome of the game.

#### 4.6.2 Corollary 2

An increase in the return on resource investment can enhance both parties' willingness to form the consortium.

By taking the partial derivatives of Equation (15) with respect to *α* and *β*, we obtain Equations (18) and (19):

$$\frac{\partial S_{\mathrm{ADBC}}}{\partial \alpha }\;=\;\frac{C_{1}}{2\lambda \theta C_{2}}\;>\;0$$ (18)

$$\frac{\partial S_{\mathrm{ADBC}}}{\partial \beta }\;=\;\frac{C_{2}}{2\lambda \theta C_{1}}\;>\;0$$ (19)

Analysis shows that S_ADBC_ is a monotonically increasing function of *α* and *β*; that is, as *α* and *β* increase, S_ADBC_ gradually grows. Whether *α* and *β* increase individually or jointly, S_ADBC_ increases, indicating that higher returns on resource investment promote the formation and consolidation of the TCM consortium. In other words, if joining the consortium yields higher returns relative to the costs incurred, the willingness to form the consortium will be strong, and the probability that the game system evolves toward the (cooperation, cooperation) strategy will be higher.

Similarly, by taking the partial derivatives of Equation (15) with respect to *λ*, *θ*, C_s1_, C_s2_, C_r_ and *R*, the following conclusions can be derived.

#### 4.6.3 Corollary 3

The greater the resource complementarity between the two parties, the higher the likelihood that they will form a consortium and engage in joint activities.

#### 4.6.4 Corollary 4

The closer the overall strength between the two parties, the lower the likelihood of free-riding by the weaker party and excessive appropriation of surplus by the stronger party, and consequently, the higher the probability that both parties will form a TCM consortium for joint collaboration.

#### 4.6.5 Corollary 5

The greater the cost savings C_s1_ and C_s2_ resulting from the involvement of a third party, the higher the willingness of the leader and the collaborative partner to form a TCM consortium.

#### 4.6.6 Corollary 6

The higher the service fee C_r_ paid to third-party service providers, the lower the likelihood that the core firm and its partners will form a collaborative alliance. Third-party services are necessary, and it is reasonable for providers to charge appropriate fees. However, excessively high fees may reduce the probability of forming a TCM consortium and adversely affect its stable operation.

#### 4.6.7 Corollary 7

As the penalty for breach of contract *R* increases, the probability that the core enterprise and its partners will form a consortium and engage in collaborative innovation gradually increases.

In summary, the resource input costs, rates of return, degree of resource complementarity, and relative comprehensive strength of the various innovative entities within a TCM consortium all exert significant influence on member selection behavior. The higher the returns on resource inputs, the stronger the degree of resource complementarity, and the closer the match in comprehensive strength, the greater the probability of forming a consortium. Under such conditions, relationships among consortium members become more cohesive, which is also more conducive to the stable operation of the consortium.

When selecting partners, innovative entities within a TCM consortium are constrained by their own conditions and resources and therefore possess limited and imperfect information about potential partners. The information they obtain may be selectively presented or embellished by the other party. As a result, identifying partners with a high degree of resource complementarity and comparable comprehensive strength becomes more difficult and entails higher search and transaction costs. In this context, the introduction of a third party is necessary to provide innovative entities with accurate and reliable information.

With the involvement of third-party actors, the costs saved by innovative entities, the penalties imposed for contract breaches, and the service fees charged by third parties all affect member selection behavior within the TCM consortium. Efficient services, valuable information, and timely supervision provided by third parties can increase the likelihood of collaborative engagement among innovative entities. At the same time, sufficiently high breach penalties and reasonable third-party service fees can also promote collaborative innovation and enhance the stable operation of the TCM consortium.

### 4.7 Numerical simulation

#### 4.7.1 Initial setup

**Figure 5:**
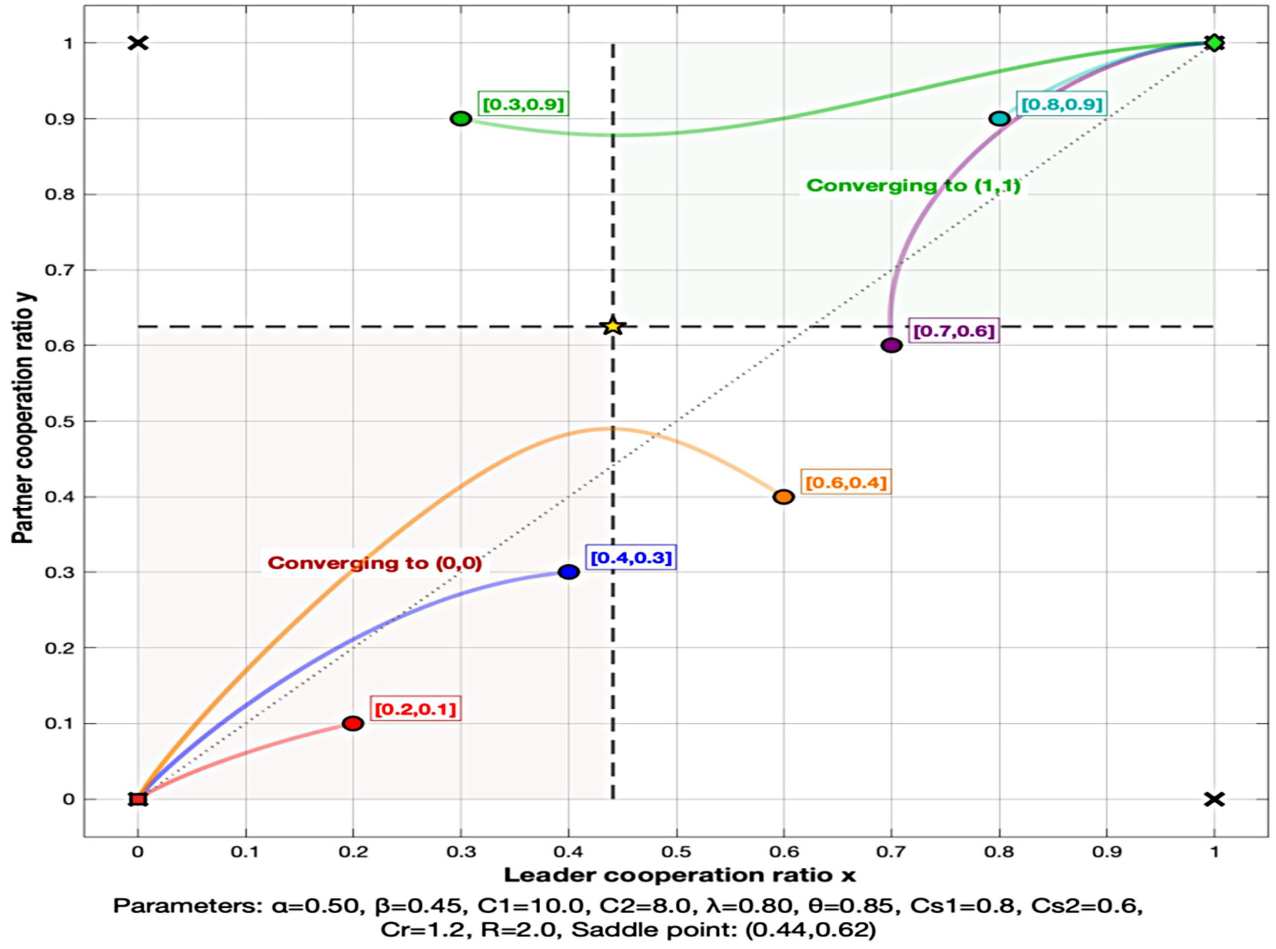
System evolution under initial conditions.

To investigate the effects of relevant parameters on the strategy choices of participating agents in the game model, and to provide a more intuitive description of the behavioral trends of these agents, the model was simulated numerically using Matlab R2020b. The parameters were set as follows: *α* = 0.5, *β* = 0.45, C_1_ = 10.0, C_2_ = 8.0, *λ* = 0.8, *θ* = 0.85, $C_{s1}=0.8$, $C_{s2}=0.6$, $C_{r}=1.2$, R = 2.0. The initial conditions [x, y] were set as six pairs: [0.2, 0.1], [0.3, 0.9], [0.4, 0.3], [0.6, 0.4], [0.7, 0.6], and [0.8, 0.9]. The system evolution results are presented in Figure 5.

Based on Figure 5, under the given parameter settings, the evolutionary process of both parties in the TCM consortium converges to one of two possible states: (0, 0) or (1, 1). In the figure, the saddle point is located at (0.44, 0.62). The six initial conditions are distributed across different regions, and their evolutionary trajectories illustrate the influence of the initial cooperation willingness on the final stable strategies. When [x, y] are assigned different initial values, the system evolution tends toward different strategy combinations, which is consistent with the discussion in Figure 4.

The initial cooperation proportion reflects the propensity of both parties to form a consortium, which is closely related to their strategic objectives and planning. In general, higher initial values drive the system to evolve more toward the (cooperation, cooperation) strategy, indicating a greater likelihood that both parties will establish a consortium.

#### 4.7.2 Effects of parameter variations on system evolution

**Figure 6:**
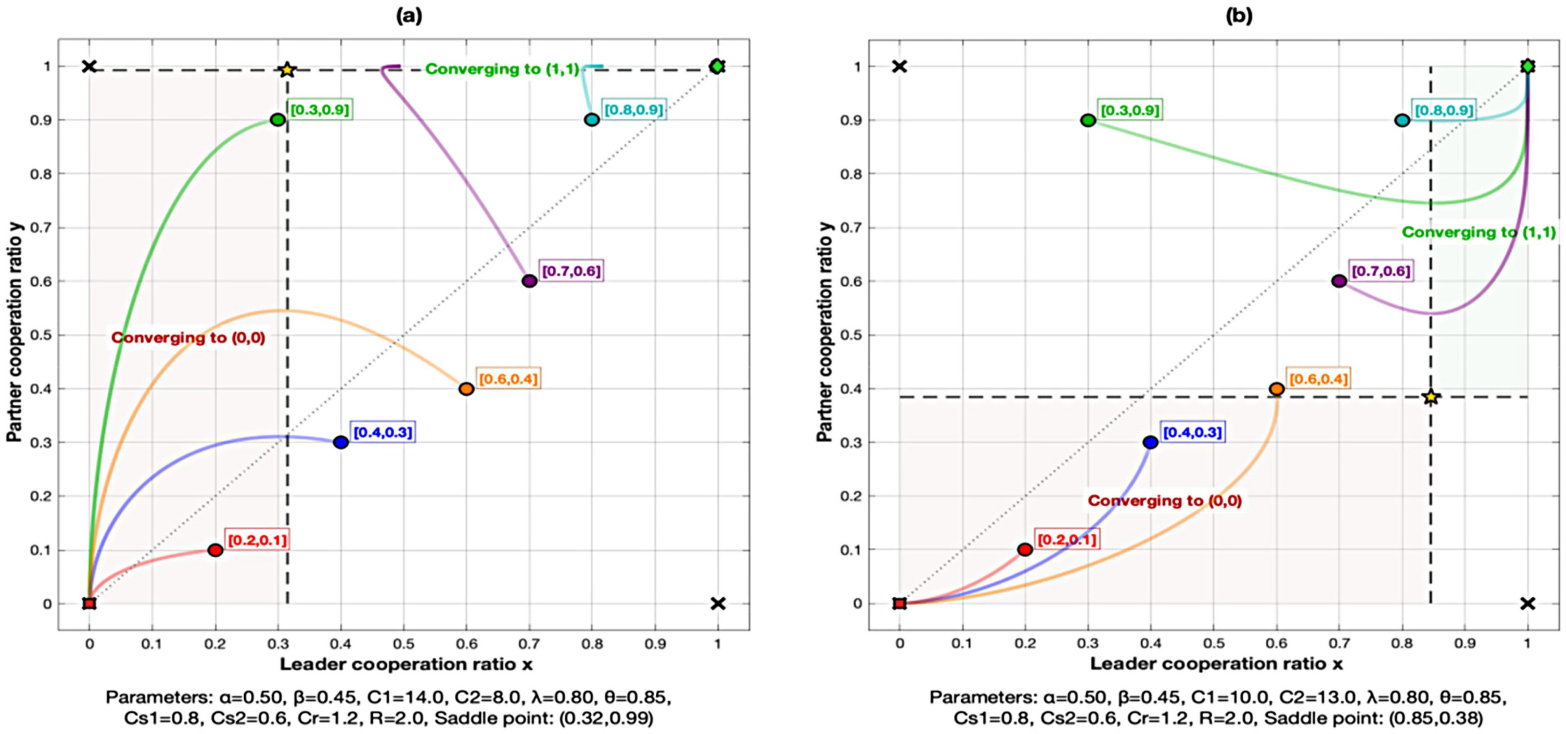
Impact of the investment costs of both players on system evolution. (**a**) Effect of C1; (**b**) Effect of C2.

With the initial conditions held constant, each simulation modifies only one parameter while keeping all other parameters unchanged. The corresponding evolution results are presented in Figures 6 through 11.

C_1_ increases from 10 to 14, and C_2_ increases from 8 to 13. The system evolution is shown in Figures 6a,b.

Compared with Figures 5, 6 shows that an increase in input costs accelerates the system's convergence toward (0, 0) while slowing its convergence toward (1, 1). This implies that the greater the resources and costs invested by both parties in forming a consortium, the more it may hinder consortium formation, indicating that input costs have a negative effect on cooperation between the two parties.

**Figure 7:**
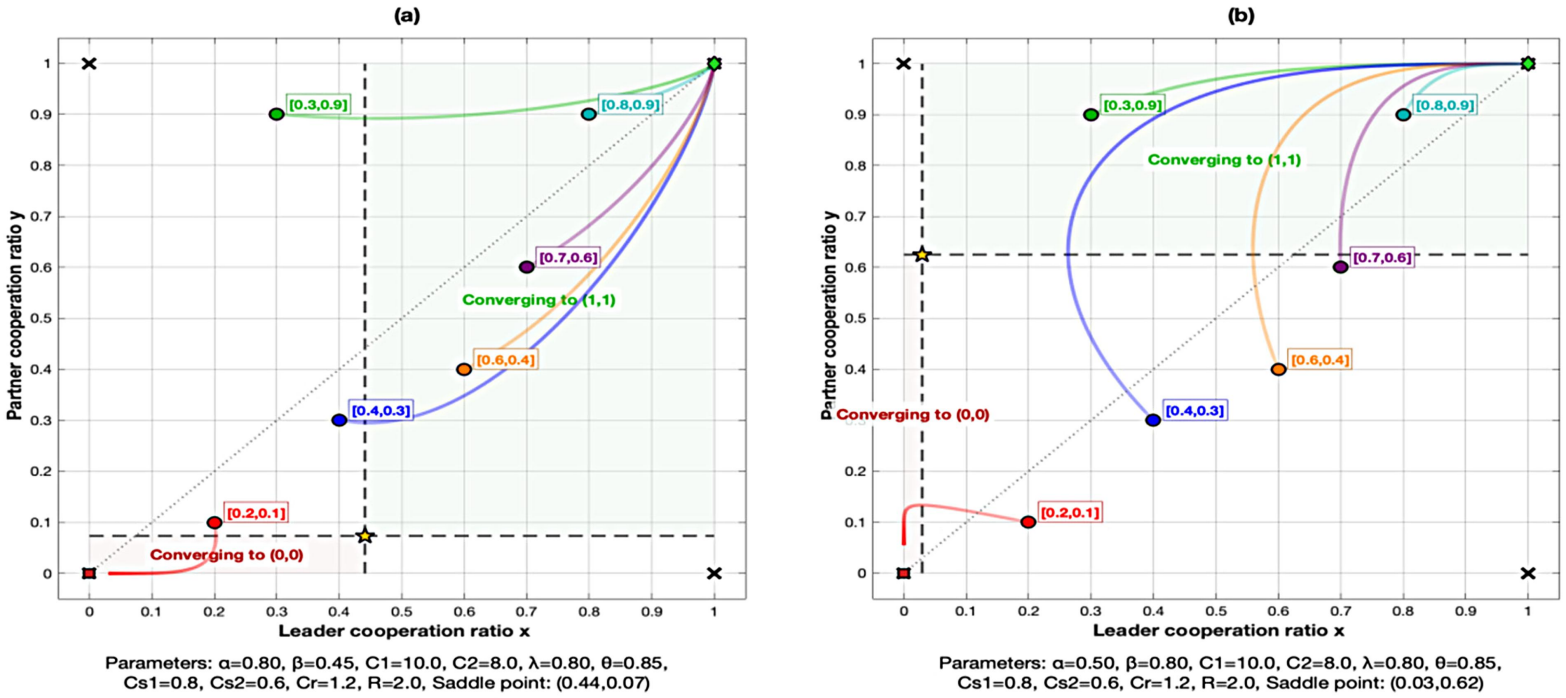
Impact of the investment costs of both players on system evolution. (**a**) Effect of *α*; (**b**) Effect of *β*.

*α* increases from 0.5 to 0.8, and *β* increases from 0.45 to 0.8. The system evolution is shown in Figures 7a,b.

**Figure 8:**
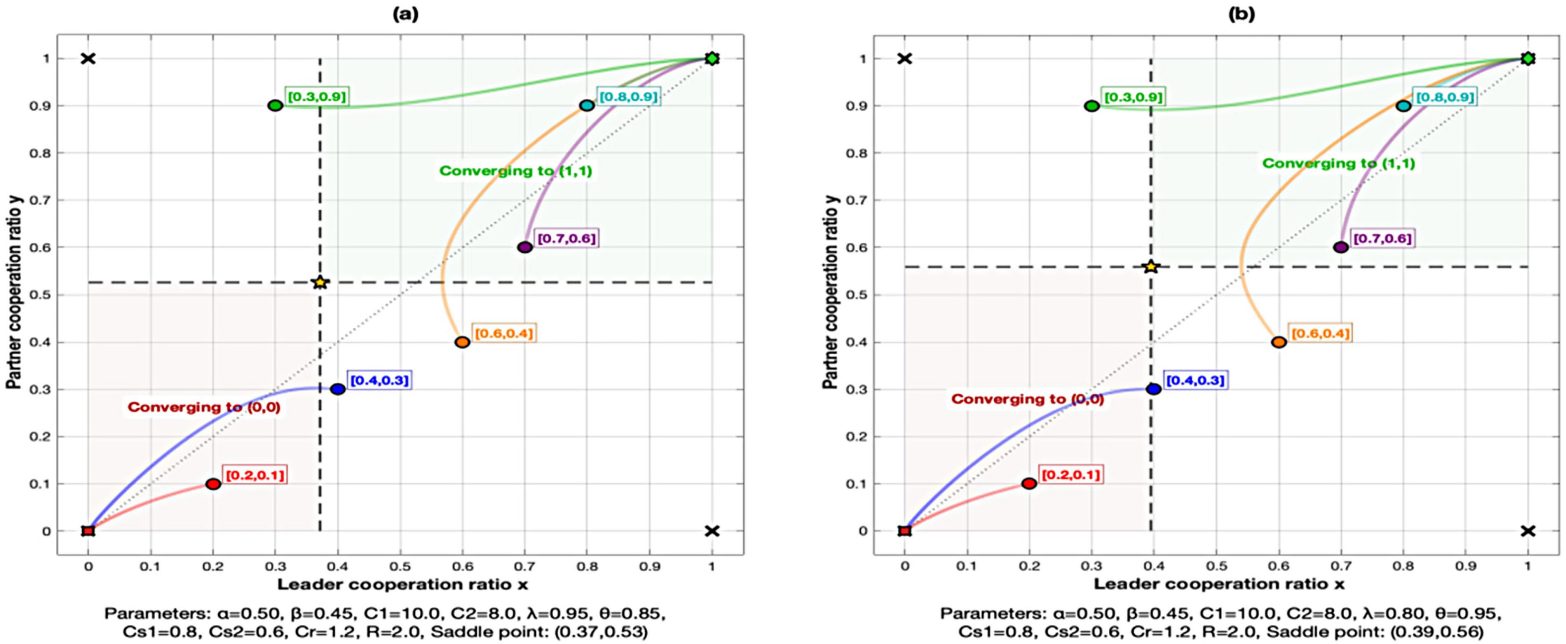
Impact of the investment costs of both players on system evolution. (**a**) Effect of *λ*; (**b**) Effect of *θ*.

*λ* increases from 0.8 to 0.95, and *θ* increases from 0.85 to 0.95. The system evolution is shown in Figures 8a,b.

**Figure 9:**
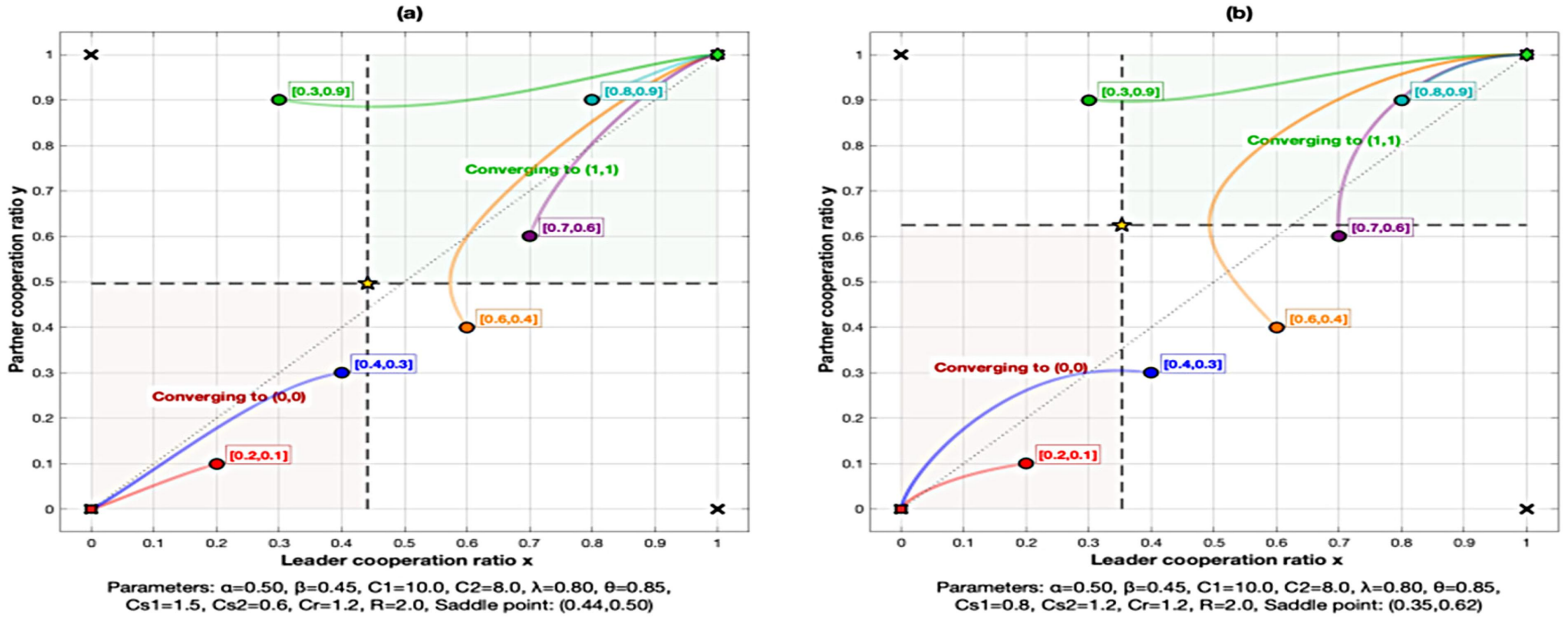
Impact of the investment costs of both players on system evolution. (**a**) Effect of Cs1; (**b**) Effect of Cs2.

C_s1_ increases from 0.8 to 1.5, and C_s2_ increases from 0.6 to 1.2. The system evolution is shown in Figures 9a,b.

Compared with Figures 5, 7–9 show that increases in cooperation benefits, resource complementarity, relative strength between the two parties, and cost savings all significantly promote the system's convergence toward (1, 1). Moreover, if the initial willingness to cooperate is high, the convergence process becomes faster. For example, when the two parties have comparable strength—such as firms with strong market expansion capabilities and research institutions with substantial technological accumulation—the likelihood of forming a consortium is significantly increased.

**Figure 10:**
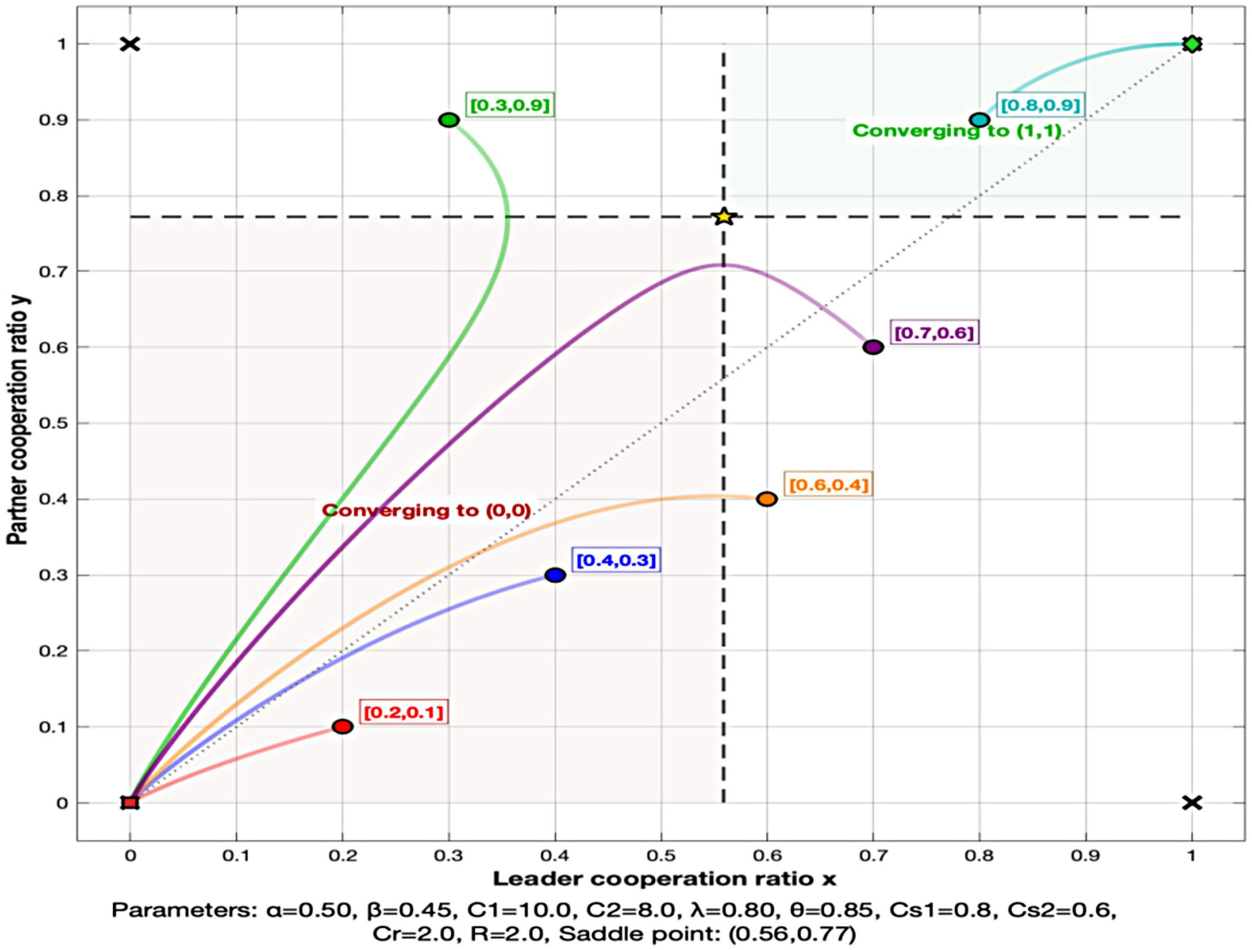
Impact of third-party benefit returns on system evolution.

C_r_ increases from 1.2 to 2.0. The system evolution is shown in Figure 10.

Compared with Figures 5, 10 shows that the benefits obtained by third-party service providers significantly influence the direction of system convergence, increasing the tendency toward (0, 0). This implies that although the involvement of third-party actors can help regulate the formation and operation of consortia, they do not directly create economic value, and their gains are derived from other consortium members. As a result, their participation reduces the likelihood of consortium formation.

**Figure 11:**
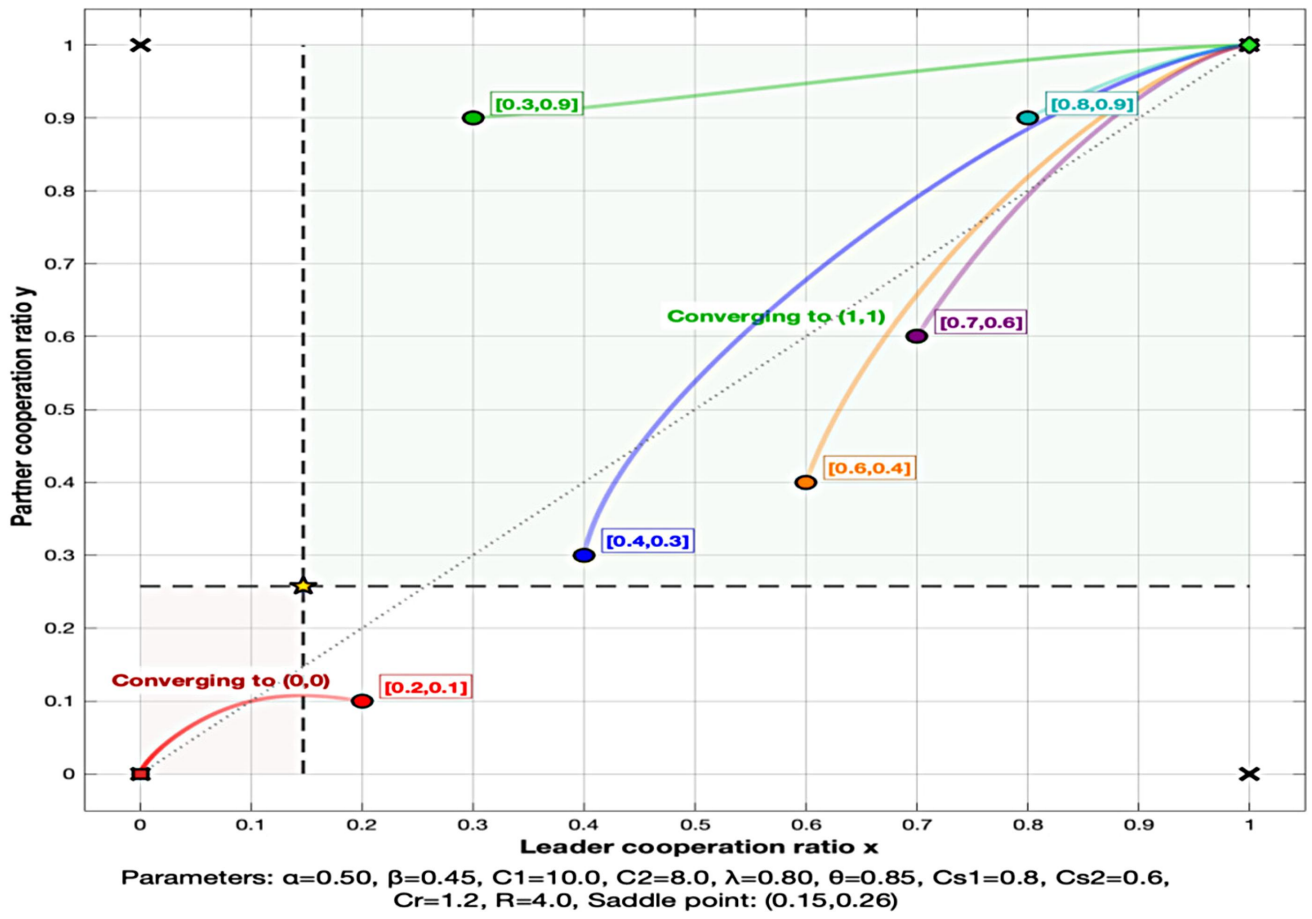
Impact of default penalties on system evolution.

R increases from 2.0 to 4.0. The system evolution is shown in Figure 11.

Compared with Figures 5, 11 shows that as the penalty for breach increases, both the speed and the likelihood of convergence toward (1, 1) are enhanced. This indicates that when high penalties are imposed for unethical behaviors such as intellectual property leakage and free-riding, members are more inclined to engage in honest cooperation to avoid punishment, thereby increasing the probability of consortium formation.

The above simulation results further validate the analyses of Corollary 1 through 7. For example, increases in the resource complementarity coefficient (*λ*), the comparative strength parameter (*θ*), and default penalties (R) all increase the likelihood of forming cooperation. In addition, third-party involvement affects cooperation: cost savings (C_s1_, C_s2_) encourage cooperation, while excessively high service fees (C_r_) discourage it. These findings offer important guidance for member selection and mechanism design in the practical operation of TCM consortia.

## 5 Policy recommendations for constructing the member selection mechanism of TCM consortia

### 5.1 The role of breach penalties in the formation and stability of TCM consortia

In collaborative innovation, the pursuit of self-interest maximization may give rise to opportunistic behavior. Opportunistic actors prioritize unilateral gains over reciprocity, seeking to appropriate knowledge and technologies from their partners and, once successful, withdrawing from the collaboration to independently exploit the acquired resources for further benefit (33).

In the context of member selection within TCM consortia, the establishment of reasonable breach penalties can effectively curb such opportunism and ensure the orderly conduct of joint innovation activities.

All participating innovative entities should operate under effective contractual constraints. Breach penalty clauses constitute an important component of consortium agreements and, when properly designed and enforced, can deter opportunistic defection and reduce the costs imposed on cooperating parties, thereby sustaining incentives for consortium formation and continuity (34). Losses arising from breach typically include not only sunk costs associated with prior resource investments but also implicit costs stemming from knowledge spillovers and technology diffusion—issues that are particularly salient for TCM enterprises given the tacit and cumulative nature of traditional medical knowledge (15).

TCM enterprises participating in consortia are required to share core intangible assets, including traditional processing techniques, origin-based processing methods for geo-authentic medicinal materials, key compatibility principles underlying classical prescriptions, and proprietary secret formulas. Such forms of knowledge are typically protected as trade secrets rather than through formal intellectual property rights. Once acquired by a collaborating party and independently patented, the originating enterprise may incur substantial losses.

Accordingly, consortium agreements should incorporate targeted penalty provisions addressing the unauthorized disclosure or appropriation of these core TCM technologies. These may include, for example, compensation significantly exceeding standard breach damages, the forfeiture of any future rights to use the misappropriated knowledge, and the disclosure of breach records within relevant industry circles. By explicitly defining protected assets in terms such as “proprietary TCM formulas” and “traditional processing techniques,” breach penalty clauses can deter opportunistic behavior and mitigate the hidden costs associated with knowledge spillovers.

Two considerations are critical in designing breach penalties. First, penalties should not be set too low. Insufficient penalties fail to deter opportunistic behavior and may reduce overall cooperation, as shown in voluntary public goods games with shared punishment. Second, breach penalties should be estimated with as much precision as possible. Since contracts are concluded prior to the commencement of joint innovation, penalty levels are inevitably based on negotiated expectations and experiential judgment. If penalties prove inadequate to cover actual losses when a breach occurs, affected parties may subsequently adjust their strategies defensively—by reducing resource inputs, shortening collaboration horizons, prioritizing short-term returns, or adopting overly aggressive yet immature innovation paths.

From an international perspective, this finding resonates with global experiences in collaborative R&D governance, where credible enforcement mechanisms are essential for sustaining cross-organizational and cross-border cooperation. For China, refining breach penalty mechanisms in TCM consortia offers a pathway to enhance contractual credibility domestically, while also aligning with international best practices in innovation governance. Moreover, China's experience in managing collaboration around knowledge-intensive and culturally embedded resources such as TCM can provide valuable lessons for other countries seeking to regulate cooperative innovation in traditional knowledge–based industries.

### 5.2 The role of collaborative intentions and innovation goal alignment in TCM consortium formation

The likelihood of forming a TCM consortium increases when innovative entities hold optimistic expectations regarding the returns from collaborative innovation and exhibit strong willingness to cooperate. Such collaborative intent manifests in several key dimensions.

First, the relative balance of comprehensive capabilities among potential partners matters. When disparities in technical strength, capital capacity, or market access are limited, parties are more inclined to engage in cooperation on an equal footing (20).

Second, prior experience with successful consortia enhances credibility. Entities—particularly TCM enterprises—that have previously participated in effective collaborative arrangements are more readily accepted as partners, as demonstrated success builds trust and reduces perceived cooperation risks (35). Enhanced trust, in turn, lowers the probability of breach and contributes to both the formation and long-term stability of TCM consortia.

Third, the existence of innovation projects aligned with market demand is crucial. Joint innovation should not be insulated from market realities; projects lacking commercial viability are unlikely to generate favorable return expectations, thereby weakening collaborative incentives. This consideration is especially relevant for TCM enterprises operating in increasingly competitive global markets, where regulatory standards, consumer preferences, and evidence-based validation play a growing role (36).

Fourth, the degree of complementarity among innovation resources is decisive. Higher complementarity—such as between clinical experience, pharmaceutical manufacturing capacity, and academic research expertise—enables more efficient resource utilization and strengthens partners' motivation to collaborate (37).

Equally important is the rational design of innovation objectives. Objectives must be scientifically grounded and realistically reflect the scale of resource commitments, the distribution of innovation risks, and the expected allocation of benefits (38, 39). At the core of these objectives lies the anticipation of innovation returns, which, in the evolutionary game framework, are represented by the parameters *α* and *β* (see Section 4.3). In addition to these modeled resource input returns, expectations regarding the market translation and commercialization of joint innovation outcomes play a critical role in guiding sustainable collaboration. Accurate market forecasting and regulatory assessment—particularly in relation to international pharmaceutical standards—are essential for aligning innovation objectives with feasible resource commitments.

A typical form of resource complementarity in the TCM context can be observed in collaborations among tertiary hospitals, pharmaceutical firms, and research institutions. The goal alignment needs to be specified in relation to the distinctive stages of TCM innovation. For instance, where the objective is to develop a new drug derived from a classical prescription, the innovation agenda should explicitly cover the authentication of medicinal materials and their quality standardization, the integration of traditional processing techniques with modern extraction methods, and the construction of an evidence base that meets international regulatory requirements (e.g., those of the FDA or the EU framework for traditional herbal medicines).

In the TCM context, these expected returns must account for long development cycles and heightened regulatory uncertainty. In addition, for product categories with strong TCM specificity—such as herbal granules and compound preparations based on classical prescriptions—goal alignment should also ensure consistency with the standards of the Chinese Pharmacopoeia as well as relevant international guidelines for botanical drug registration.

### 5.3 The role of third-party participation in the governance of TCM consortia

The participation of third-party actors—whether governmental or non-governmental—can significantly enhance the efficiency and effectiveness of member selection in TCM consortia. Third parties can provide reliable and high-quality information, reduce search and matching costs, and assume roles in coordination, supervision, and dispute resolution (23, 40). They may also impose sanctions on opportunistic behavior, thereby reinforcing contractual discipline.

Although government agencies typically do not participate directly in the formation of TCM consortia, their supportive role is critical. Through policy guidance, regulatory facilitation, and institutional endorsement, governments can create a favorable environment for consortium formation and stable operation (41). Non-governmental actors, by contrast, may either participate as consortium members—contributing specialized services and sharing in innovation outcomes—or function as market-oriented intermediaries, offering information and coordination services in exchange for service fees (26).

The involvement of third parties can substantially reduce the information asymmetries and transaction costs faced by core innovative entities, particularly enterprises that often lack comprehensive visibility into potential partners' true capabilities (42). However, third-party participation entails costs, and the level of service fees must be carefully calibrated. Reasonable fees support efficient member selection and coordination, whereas excessive charges may deter participation and impede consortium formation.

In the context of TCM consortia, third-party participation should be directed toward addressing information asymmetries and evaluation challenges. For example, when enterprises screen patents from universities or research institutes, they often face difficulties in assessing the stability of medicinal material quality, the reliability of claimed clinical efficacy, and the extent to which processing techniques can be scaled for industrial production (43).

A useful reference can be found in the third-party scientific assessment bodies established under the EU framework for botanical drug registration. Specialized third-party evaluation institutions tailored to the TCM sector could be introduced, with functions such as: quality authentication of medicinal materials (e.g., geo-authenticity verification through DNA barcoding and consistency assessment of active ingredient content), independent evaluation of clinical efficacy (e.g., validation of classical prescriptions based on real-world data), and assessment of the pilot-scale feasibility of technological outcomes.

Such institutions can significantly reduce firms' trial-and-error costs, helping to avoid situations in which substantial resources are invested in acquiring patents that ultimately fail to meet regulatory approval requirements or cannot be translated into stable production.

In addition, the pricing of third-party services should be calibrated to the average return profile of TCM innovation projects, so as to avoid excluding small and medium-sized enterprises due to excessive fees.

From a global standpoint, the role of intermediaries and public institutions in fostering collaborative innovation is widely recognized. China's evolving practices in leveraging third-party support for TCM consortia provide a useful case for understanding how state and market actors can jointly facilitate cooperation in knowledge-intensive sectors.

## 6 Conclusion, research limitations and future directions

Technological innovation serves as the driving force for enhancing the overall competitiveness of the TCM industry. A key determinant of improving innovation capability lies in the efficient utilization of available innovation resources. Forming TCM consortia that bring together enterprises, academic and research institutions, intermediary agencies, and financial entities represents a practical and promising approach for TCM companies.

By modeling member selection in TCM consortia as an evolutionary game under bounded rationality, this study demonstrates that the stability and success of the consortium depend not solely on one-off optimal decisions, but on the dynamic interactions among payoff expectations, contractual enforcement, resource complementarity, and institutional support. Specifically, higher returns on resource investment, stronger complementarity of innovation resources, closer alignment of comprehensive capabilities, reasonable breach penalties, and the involvement of third-party actors significantly increase the likelihood that members adopt cooperative strategies and sustain stable collaboration. Conversely, excessive third-party service fees or misaligned resource allocations may hinder consortium formation and destabilize existing alliances.

Appropriate member selection not only ensures access to complementary innovation resources but also facilitates timely communication and mutual trust, thereby reducing resource wastage and improving both operational efficiency and innovation outcomes. These findings suggest that consortium formation is a continuous and adaptive process shaped by the evolving strategic interactions of its members.

From an international perspective, experiences in other knowledge-intensive and multi-actor innovation sectors offer valuable lessons for TCM consortia in China. Conversely, China's practice of structuring consortia around boundedly rational actors, evolutionary dynamics, and well-calibrated incentives may also provide useful insights for countries seeking to organize collaborative innovation in traditional knowledge or natural product industries.

Nevertheless, several limitations should be noted. First, the model is developed under bounded rationality with a simplified strategy space, which may not fully capture the complexity of real-world behaviors, policy adjustments, and market dynamics. Second, it is important to emphasize that this study adopts a theoretical modeling approach. In this context, analytical rigor is primarily reflected in the coherence of the theoretical framework, the internal consistency of the model, and the logical derivation of strategic outcomes, rather than in empirical estimation. This methodological orientation inevitably limits direct validation using large-scale observational data.

At the same time, this limitation also points to a valuable empirical research agenda. In practice, collaboration data among TCM enterprises, particularly information related to knowledge sharing, contractual penalties, and benefit allocation, is often highly sensitive and difficult to access, which partly explains the current lack of quantitative evidence in this domain. Future studies could address this gap by drawing on qualitative and mixed-method approaches, such as in-depth case analyses of representative TCM innovation consortia or semi-structured interviews with key participants, in order to obtain fine-grained data on parameters such as collaboration duration, penalty intensity, and resource contribution structures. Such efforts would enable more precise calibration of the model and strengthen the empirical relevance of evolutionary game analysis in this context, thereby bridging the gap between theoretical modeling and the real-world governance of TCM innovation consortia.

## Data Availability

The original contributions presented in the study are included in the article/Supplementary Material, further inquiries can be directed to the corresponding authors.
